# Prevalence and clinical characteristics associated with peripheral neuropathy amongst persons on HAART in Busia County, Kenya

**DOI:** 10.4102/sajp.v76i1.1430

**Published:** 2020-08-25

**Authors:** John N. Mukoma, Joseph M. Matheri, Nassib Tawa

**Affiliations:** 1 Department of Physiotherapy, Busia County Referral Hospital, Busia, Kenya; 2 Department of Physiotherapy, College of Health Sciences, Jomo Kenyatta University of Agriculture and Technology, Nairobi, Kenya; 3 The Center for Research in Spinal Health & Rehabilitation Medicine, Nairobi, Kenya

**Keywords:** clinical symptoms, HAART, HIV-related peripheral neuropathy, CHANT, physiotherapy

## Abstract

**Background:**

Despite improved immunological and viral load control, the prevalence of HIV/AIDS-related peripheral neuropathy among survivors on highly active antiretroviral therapy (HAART) is rising globally raising public health concerns.

**Objectives:**

To determine the prevalence and clinical characteristics of peripheral neuropathy amongst persons on HAART attending Comprehensive Care Clinics in Busia County, Kenya.

**Method:**

This cross-sectional descriptive quantitative study utilised purposive sampling and included 289 adults living with HIV/AIDS. Data collection was undertaken using the Clinical HIV Associated Neuropathy Tool (CHANT) and analysed using the Statistical Package for the Social Sciences version 25.0.

**Results:**

Of people on HAART, 68.17% (197 amongst 289) had peripheral neuropathy. The majority were female 76.8% (*n* = 222), 38.1% (*n* = 110) were between 41 and 50, and 35% (*n* = 101) were widowed. The most common primary symptom was reduced right foot big toe vibration (76.8%, *n* = 222). There was a strong positive relationship (*r* = 0.621, *P* = 0.000) between foot vibration and illness. There was a statistically significant influence of demographic characteristics of persons on HAART on PN as they accounted for 98.5% of the variance (*R*^2^ = 0.985).

**Conclusion:**

Peripheral neuropathy is prevalent and is significantly influenced by socio-demographic characteristics of persons on HAART-PN. Early diagnosis and exercise guidance by physiotherapists is key in forestalling severe symptoms, disability and poor quality of life.

**Clinical implications:**

There is need to screen persons living with HIV on HAART for PN to establish their medical, physiotherapy and rehabilitation needs. Early diagnosis will encourage healthcare workers to start interventions to prevent progression of impairment, onset of disability and decrease in quality of life. Therefore, adaptation of PN screening tools and physiotherapeutic interventions should be considered.

## Introduction

The prevalence of peripheral neuropathies amongst seroreactive persons is increasing globally raising public health concerns (Aminoff & Portegies 2007; Choi et al. 2011; Wulff, Wang & Simpson 2000). Peripheral neuropathy (PN) is defined as the presence of either a pain or burning sensation, aching or numbness combined with the absence of reflexes or impairment resulting from a vibration sensation in the great toe (Evans et al. 2011). Globally, between 30% and 60% of individuals with HIV are diagnosed with neuropathic conditions. With the improved use of chemoprophylaxis highly active antiretroviral therapy (HAART), the prevalence of HIV-related neuropathies resulting from HIV/AIDS has greatly changed (Evans et al. 2011; Ferrari et al. 2006; Mehta et al. 2011). In a study to determine the outcomes of HIV infection on the nervous system, Aminoff and Portegies (2007) noted that although there was a decline in the incidence of HIV-related neuropathies, the prevalence of neurological impairments and disability has increased significantly due to the improved survival of persons with HIV/AIDs accessing and using HAART.

Though seldom seen in children, PN is the commonest neurological problem amongst adults who are infected with HIV (Colloca et al. 2017; Keltner et al. 2012). Tumusiime et al. (2014) noted that PN currently affects persons living with HIV as a complication of both HIV and ART.

Whereas HIV associated ‘distal symmetrical polyneuropathy’ is a common neurological problem, variable incidences of its occurrences, ranging from 19% to 66%, are reported (Afzal et al. 2015). Moreover, HIV-associated peripheral neuropathies are often misdiagnosed or the diagnosis is overlooked (Wulff et al. 2000).

There is also a growing body of evidence linking the use of antiretroviral agents (HAART) to neuropathic diseases (Attal & Bouhassira 2015). For instance, Wulff et al. (2000) noted that distal symmetrical polyneuropathy is a frequent type of PN reported amongst persons with HIV infection who have neurotoxic levels of antiretroviral agents or advanced immunosuppression. In the USA, where HAART is readily available, the greatest proportion of the neuropathic disease burden is PN and HIV-associated cognitive dysfunction (Ellis et al. 2010). In contrast, in low-to-middle income countries where HAART is not readily available, opportunistic infections of the central nervous system account for the greatest proportion of the reported neuropathic disease burden (Kongsiriwattanakul & Suankratay 2011). In a cross-sectional study, conducted in Bangkok, Thailand, to determine the frequency of sensory neuropathy amongst 118 HIV-positive patients, Sithinamsuwan et al. (2008) found a 28% possibility of sensory neuropathy amongst those not on HAART. Cashman and Hoke (2015) observed that regardless of the improved control of the virus in the past decade, the incidence of neuropathy amongst HIV/AIDS patients significantly increased from 13% in 1993 to 50% in 2015.

Most of these studies were conducted in developed countries including the USA, and the UK (Aminoff & Portegies 2007; Evans et al. 2011). In Central Africa, the first verifiable case of HIV/AIDS was reported in 1983, as noted by Song’ony (2008). The spread of HIV/AIDS has been blamed on globalisation including migration, trade and travel (Todrys & Amon 2009). Civil unrest in countries like Uganda, the Democratic Republic of Congo (DRC), and South Africa is also believed to have brought HIV/AIDS to new locations as a result of large-scale migration (WHO 2018). Unlike in the USA and other European countries, African victims have a higher incidence of transmittance of the virus through heterosexual intercourse than from homosexuality or drug addiction (Song’ony 2008). In sub-Saharan Africa, little information is available concerning the impact of HIV/AIDS-related neuropathy, ensuing disability and the quality of life challenges amongst survivors of HIV. However, in a cross-sectional study, involving 800 HIV-positive participants, conducted in Uganda, to assess risk factors, prevalence, and neuropathies-related disabilities, Saylor et al. (2017) found a 19% prevalence rate of peripheral neuropathy amongst them. Therefore, the aim of our study was to determine the prevalence, and the clinical characteristics associated with PN amongst persons on HAART attending a Comprehensive Care Clinic in Busia County, Kenya. The information generated from our study on the burden and clinical characteristics associated with PN could possibly contribute to practice, implementation of health and disability policies including planning and provision of rehabilitation services.

## Method

Our study was undertaken at the Comprehensive Care Clinics (CCC) in Busia County one of the 47 counties in Kenya. In the 2009 National Census, Busia County had an estimated population of 823 504 which is 1.9% of the national population, with most of the population being female (52%). The county’s poverty level was 64.2% compared to the National poverty level of 45.9%. It had an estimated 164 701 households with an average family size of 5 and a literacy level of 75.3% amongst those aged 16 years and above.

Our cross sectional study utilised quantitative methods for data collection. We considered the population of all adults of both genders aged 20 to 68 years, with HIV infection and on HAART, who were living in Busia County. The Kenya HIV county profiles (2016) reported that Busia County had 38 549 people infected with HIV/AIDS. According to the same report, there were more female persons (8.3%) than male (5.0%). Persons on HAART care were spread over 67 CCC. According to the 2018 Kenya guidelines for treating and preventing HIV, patients testing positive for HIV are given AZT+3TC+EPV or RAL, ABC +3TC +LPV/r, AZT +3TC +EPV (or RAL), TDF + 3TC +ATV/r, AZT + 3TC +DTG (or EPV), TDF + 3TC + ATV/r^2^ as either first- or second-line HAART treatments.

A simple random technique was used to select study sites from the 67 CCC, which resulted in four sites being selected. Further, purposive sampling conducted in the selected sites resulted in 300 potential participants on HAART being recruited between the months of March and April 2019.

A piloted version of the validated Clinical HIV Associated Neuropathy Tool (CHANT) was used to assess symptoms (pain, numbness, ankle reflexes and vibration sense) to establish the presence of PN amongst persons living with HIV and on HAART. Piloting was done to assess the user friendliness of the CHANT tool before adopting it. According to the developers, the CHANT tool was validated clinically and field tested on seropositive clients in the UK and in Johannesburg, South Africa (Woldeamanuel et al. 2016).

In both the South African and the UK samples it recorded good sensitivity = 74%, specificity = 85%, internal consistency = 0.88 (Cronbach’s alpha) and inter-item correlation = 0.73 (Spearman’s Rho) and inter-rater agreement > 0.93 (Spearman’s Rho).

### Statistical analysis

Data were entered into MS Excel and imported to the Statistical Package for Social Sciences (SPSS) software for analysis. Descriptive statistics for demographic variables (including gender, level of education, marital status and illness) were calculated based on the frequencies and percentage distributions. A correlation analysis using Pearson’s product-moment correlation (*r*) was used to analyse the relationship between the demographic characteristics and prevalence distribution of PN. The statistical influence of demographic characteristics on PN amongst persons on HAART was further analysed by an analysis of variance as well as a linear regression analysis at a significance level of *p* < 0.005.

### Ethical consideration

The study obtained ethical clearance from Jomo Kenyatta University of Agriculture and Technology (JKUAT) Ethical Review Committee (REF: JKU/2/4/896B) and was approved by the National Commission of Science, Technology and Innovation (NACOSTI/P/19/146317/29284).

## Results

Out of the 300 questionnaires administered, 289 were completed correctly whilst 11 had incomplete data and were discarded. This resulted in a 96.33% response rate. Of the 289 respondents, 76.8% (*n* = 222) were female. Thirty-eight (38.1% *n* = 110) were middle-aged adults between 41 and 50, followed by those-aged 51 and above, 36% (*n* = 104). Additionally, 35% (*n* = 101) were widowed whilst only 28% (*n* = 81) were married. The majority of the respondents, 53.6% (*n* = 155), had primary school education, and 27.7% (*n* = 80) had secondary school education (Table 1).

**TABLE 1:** Demographic characteristics of persons on HAART (*n* = 289).

Demographic variables	Characteristics	Frequency	Percentage %
Gender	Male	67	23.2
	Female	222	76.8
	**Total**	**289**	**100.0**
Age	≤ 20 years	10	3.5
	21–30 years	18	6.2
	31–40 years	47	16.3
	41–50 years	110	38.1
	≥ 50 years	104	35.9
	**Total**	**289**	**100.0**
Education	No formal school	47	16.3
	Primary school	155	53.6
	Secondary school	80	27.7
	Tertiary	7	2.4
	**Total**	**289**	**100.0**
Marital status	Single	32	11.1
	Married	81	28.0
	Cohabiting	42	14.5
	Separated	18	6.2
	Divorced	15	5.2
	Widowed	101	35.0
	**Total**	**289**	**100.0**
Illness	With illness	197	68.2
	Without illness	92	31.8
	**Total**	**289**	**100.0**

*Source*: Primary data

### Prevalence of peripheral neuropathy amongst males and females on HAART

Symptomatic PN had a prevalence of 68.1%% (197 of 289). Illness was independently associated with the development of PN amongst the participants. The primary signs and symptoms reported by participants with PN were reduced right foot big toe vibration (76.8%, 222 of 289), right foot pain (69.55%, 201 of 289) followed by right ankle reflex reduction (74.7%, 216 of 289). Men had right foot pain (*n* = 41, 14.19%), left foot pain 12.80% (*n* = 37), right foot numbness 15.92% (*n* = 46), left foot numbness, 15.22% (*n* = 44), reduced right foot vibration 22.83% (*n* = 66), left foot vibration 22.15% (*n* = 64), right ankle reflex reduction 23.18% (*n* = 67) and left ankle reflex reduction 21.80% (*n* = 63). Women had right foot pain (*n* = 160, 55.36%), left foot pain 53.29% (*n* = 154), right foot numbness 50.52% (*n* = 146), left foot numbness 49.13% (*n* = 142); reduced right foot vibration 53.97% (*n* = 156), reduced left foot vibration 54.33 (*n* = 157), right ankle reflex reduction 51.56% (*n* = 149) and left ankle reflex reduction 51.90% (*n* = 150) (Table 2).

**TABLE 2:** Prevalence of peripheral neuropathy amongst male and female participants on HAART.

PN diagnostic indicators	With PN		Without PN	
	*n* = 197	%	*n* = 92	%
**Male (*n* = 67)**				
Right foot pain	41	14.19	26	9.0
Left foot pain	37	12.80	30	10.38
Right foot numbness	46	15.92	21	7.27
Left foot numbness	44	15.22	23	7.96
Right foot big toe vibration	66	22.83	1	0.35
Left foot big toe vibration	64	22.15	3	1.04
Right ankle reflex	67	23.18	0	0
Left ankle reflex	63	21.80	4	1.38
**Female (*n* = 222)**				
Right foot pain	160	55.36	62	21.45
Left foot pain	154	53.29	68	23.53
Right foot numbness	146	50.52	76	26.30
Left foot numbness	142	49.13	80	27.68
Right foot big toe vibration	156	53.97	6	2.08
Left foot big toe vibration	157	54.33	5	1.73
Right ankle reflex	149	51.56	3	1.04
Left ankle reflex	150	51.90	2	0.69

*Source*: Primary data

PN, peripheral neuropathy.

### Relationship between peripheral neuropathy and demographic characteristics

The results indicate that there was some relationship between PN and demographic characteristics. The results illustrated in Table 3 show a strong positive relationship (*r* = 0.837, *p* = 0.000) between foot pain and illness.

**TABLE 3:** Relationship between demographic characteristics (*n* = 289) and peripheral neuropathy.

Characteristics	Foot pain	Foot numbness	Foot vibration	Ankle reflex
**Age**				
Pearson correlation	0.331†	0.221	0.026	0.069
Sig. (2-tailed)	0.000	0.000	0.000	0.000
*N*	289	289	289	289
**Gender**				
Pearson correlation	0.026	0.026	0.033	0.056
Sig. (2-tailed)	0.000	0.000	0.000	0.000
*N*	289	289	289	289
**Education**				
Pearson correlation	0.083	0.013	0.670‡	0.024
Sig. (2-tailed)	0.000	0.000	0.000	0.000
*N*	289	289	289	289
**Marital status**				
Pearson correlation	0.483†	0.308†	0.084	0.016
Sig. (2-tailed)	0.000	0.000	0.000	0.000
*N*	289	289	289	289
**Illness**				
Pearson correlation	0.837‡	0.896‡	0.621‡	0.541†
Sig. (2-tailed)	0.000	0.000	0.000	0.000
*N*	289	289	289	289

*Source:* Primary data

† , Correlation is significant at the 0.05 level (2-tailed).

‡ , Correlation is significant at the 0.01 level (2-tailed).

There was a weak positive relationship between age, gender, education as well as marital status and foot pain.

Similarly there was a strong positive (*r* = 0.896, *p* = 0.0001) relationship between foot numbness and illness, and the relationship between age, gender, education, marital status and foot numbness was positive but weak. There was a strong positive (*r* = 0.621, *p* = 0.000) relationship between foot vibration sense loss and illness. There was a weak positive relationship between age, gender, education as well as marital status and foot vibration sense loss. There was a moderate positive (*r* = 0.541, *p* = 0.0001) relationship between ankle reflex reduction and illness. Similarly, there was a weak positive relationship between age, gender, education as well as marital status and reduced ankle reflex. The results indicate a strong and positive relationship (*r* = 0.670, *p* = 0.00) between foot vibration and education. Thus, it can be argued that there was a strong relationship between PN (foot pain, foot numbness, foot vibration sense and ankle reflex) and illness.

Table 3 summarises the relationship between the participants’ (*n* = 289) demographic characteristics and PN.

### Influence of peripheral neuropathy amongst persons on HAART

Table 4 shows that there was a statistically significant influence on the PN domain and demographic characteristics of people on HAART as they accounted for 98.5% of the variation (*R*^2^ = 0.985).

**TABLE 4a:** Regression results of demographic characteristics and peripheral neuropathy domains. (a) Goodness-of-fit.

Model	*R*	*R* square	Adjusted *R* square	Std. error of the estimate	Change statistics				
					*R* square change	*F* change	df1	df2	Sig. *F* change
1	0.993†	0.985	0.985	0.183	0.985	0.289	10	278	0.000

*Source:* Primary data

† , Predictors, (Constant).

**TABLE 4b:** Regression results of demographic characteristics and peripheral neuropathy domains. (b) Overall significance.

Model		Sum of squares	Df	Mean square	*F*	Sig.
1	Regression	30.245	10	3.0245	3.306	0.029†
	Residual	226.585	276	0.8210	-	-
	Total	256.830	286	-	-	-

*Source:* Primary data

† , Dependent variable: Peripheral neuropathy.

**TABLE 4c:** Regression results of demographic characteristics and peripheral neuropathy domains. (c) Individual significance.

Model		Un-standardised coefficients		Standardised coefficients			95.0% confidence interval for *B*	
		*B*	Std. error	Beta	*T*	Sig.	Lower bound	Upper bound
1	(Constant)	0.129	0.148	-	0.876	0.382	0.420	0.161
	Right foot pain	0.008	0.184	0.002	0.042	0.036	0.371	0.355
	Left foot pain	0.022	0.098	0.007	0.229	0.019	0.214	0.170
	Right foot numbness	0.018	0.194	0.006	0.095	0.002	0.400	0.363
	Left foot numbness	0.024	0.098	0.008	0.248	0.004	0.168	0.216
	Right foot big toe vibration	0.497	0.202	0.051	2.46	0.004	0.896	0.099
	Left foot big toe vibration	0.407	0.124	0.045	3.27	0.001	0.162	0.652
	Right ankle reflex	0.112	0.217	0.008	0.517	0.006	0.540	0.315
	Left ankle reflex	0.322	0.126	0.031	2.550	0.001	0.073	0.570

*Source:* Primary data

Further, the overall model also reveals a statistically significant relationship between the PN domain and demographic characteristics of persons on HAART (*F* = 3.306, *p* = 0.029). The standardised regression coefficient also shows that peripheral neuropathies in persons on HAART are statistically significant. The Beta coefficient for the peripheral neuropathy domain was found to be positive and statistically significant. The regression results are summarised in Table 4.

## Discussion

The improved survival of persons with HIV/AIDS on HAART has seen an increase in the prevalence of neurological conditions (Ferrari et al. 2006; Mehta et al. 2011; Wolfe & Barohn 2002). The results of our study confirm that PN is prevalent amongst persons on HAART in Busia County. The high prevalence of PN symptoms in our sample is an indication of a growing sub-population at risk of disability and reduced quality of life (Biraguma & Rhoda 2012; Colloca et al. 2017; Keltner et al. 2012).

Disability arising from HIV has been linked to reduced physical functioning which impacts on activities of daily living in various ways (Biraguma & Rhoda 2012; Cade, Peralta & Keyser 2004; Mehta et al. 2010). The unearthing of the prevalence of PN is critical for clinical care, physiotherapy and the rehabilitation of persons with HIV on HAART in scarcely resourced settings, particularly in Kenya.

This is the first study to report the prevalence and clinical characteristics associated with PN amongst seropositive people on ART medication in Busia County which is a resource-limited setting. The high prevalence (68%) of neuropathy amongst the respondents is similar to the study by Tumusiime et al. (2014) which found a 59% prevalence of PN in Rwanda, and to studies that show a 57% (226 of 395) (Wadley et al. 2011) prevalence rate of PN amongst people on HAART. Biraguma and Rhoda in their Rwandan study (2012) found that 40.5% of participants had PN, which again is similar to our study. Other studies have found PN common (over 40%) amongst ambulatory seropositive persons (Choi et al. 2011; Mbuya et al. 1996; Nicholas et al. 2007; Smyth et al. 2007). However, in a UK study, Skopelitis et al. (2006) reported that PN affected 36% of participants, and in an Ethiopian study Shurie and Deribew (2010) found that it affected 34.5% of patients on HAART. These figures are lower than those in our study and could be attributed to the different dynamics of the participants and the measures used to collect data (i.e. clinical presentation versus electrophysiological tests).

Persons on HAART with PN mainly experienced pain, a burning sensation or aching followed by numbness, whilst the most infrequent symptoms were pins and needles. The symptoms in our population were mild (no debilitating condition) in a large number of the respondents with PN (135 out of 289). Pain and illness had a strong positive relationship (*r* = 0.837, *p* = 0.000); this implies that pain was culturally perceived as an illness which had the potential to interrupt mobility, social interactions, work or schooling and overall quality of life.

Evidence in support of this view shows that HIV-associated neuropathic pain is common amongst persons living with HIV (Keltner et al. 2012; Wallace et al., 2007) and is associated with pain catastrophising and poor adherence to prescribed medication (Lucey et al. 2011). Moreover, it is related to poor outcomes and problematic treatment choices and a decreased quality of life (Ellis et al. 2010; Keltner et al. 2012) partly due to inappropriate classification, increased drug prescriptions (Attal & Bouhassira 2015; Wolfe & Barohn 2002) and visits to physicians as well as the illness from the pain itself and aggravating conditions (Colloca et al. 2017). Individuals usually feel a combination of symptoms, such as electric-like and burning sensations and pain from trivial (non-noxious) stimulation such as light touch (Colloca et al. 2017).

Further, clinical expression of neuropathic pain results in impairment of inhibitory modulation systems, anxiety and depression (Colloca et al. 2017; Keltner et al. 2012; Malvar et al. 2015) which have been associated with sleep problems (Colloca et al. 2017; Sandoval, Runft & Roddey 2010) and opioid use disorders (Malvar et al. 2015). According to Malvar et al. (2015) factors such as opioid use disorder, older age, being female, current and past ART treatment as well as a lack of HIV suppression predict neuropathic pain (Malvar et al. 2015).

This implies that the neuropathic pain mechanism and its accompanying complications are different from inflammatory pain caused by conditions such as rheumatoid arthritis, and therefore its treatment approaches and choices should differ (Keltner et al. 2012). This notwithstanding, our findings are different from those of Konchalard and Wangphonpattanasiri (2007) who established that numbness was the dominant symptom in most or all forms of neuropathy related to HIV.

It has been suggested that pain and paraesthesia may be early indicators of the disease, whilst numbness becomes apparent as the disease advances. Severity of PN in persons on HAART can range from mild discomfort to an incapacitating disorder, causing the individual difficulty in walking or even in standing from sitting (Keltner et al. 2012; Schifitto et al. 2002). Many persons with mild PN do not suffer any symptoms, although severe symptoms mostly occur in persons with advanced immunosuppression (Simpson & Cikurel 2006). The severe symptoms, such as ageing and other comorbidities amongst persons using ART, increase the risk of disability which impacts on multiple aspects of daily living (Biraguma & Rhoda 2012; Cade et al. 2004; Mehta et al. 2010). Management of symptoms or impairments (such as neuropathic pain), using splints (Sandoval et al. 2010) and physiotherapist-supervised exercise, has shown promising outcomes (Taylor et al. 2007; Tumusiime et al. 2019). This has the potential to influence the deployment of physiotherapists and other rehabilitation professions in universal health care and practice where exercise becomes a key strategy to prevent disability and activity limitation.

A limitation of this study was that neither the key pointers of the illness progression, such as the CD4 count and viral load, nor data on medication for tuberculosis were assessed. Hence, no association between the progression of the illness, manifestation and severity of PN could be established. Our study did not look into other risk factors of PN such as diabetes or poor nutrition.

### Implications

Our study established the presence of PN clinical symptoms in a proportion of the respondents, which has implications for functional status, participation in core activity domains and quality of life. There are therefore implications for use of validated early screening tools, diagnosis and management of PN clinical symptoms and complications to mitigate functioning loss or disablement in which physiotherapists and allied rehabilitation professionals have a role to play. Further, there is an implication for physiotherapy research to generate evidence on the effects of physical exercise on clinical symptoms and complications of PN, and the scope of practice.

## Conclusion

In conclusion, PN is prevalent amongst persons on HAART and is characterised by pain, numbness, decreased ankle tendon reflexes and vibration sense of the big toe, predominantly. Whilst diagnosis of PN amongst patients on HAART remains generally clinical, other illnesses that complicate distal neuropathies should be excluded. However, early diagnosis and exercise guided by physiotherapists have a higher likelihood of success in preventing progression to impaired function, disability and poor quality of life.
