# Corrigendum: Prevalence and clinical characteristics associated with peripheral neuropathy amongst persons on HAART in Busia County, Kenya

**DOI:** 10.4102/sajp.v77i1.1615

**Published:** 2021-11-26

**Authors:** John N. Mukoma, Joseph M. Matheri, Nassib Tawa

**Affiliations:** 1 Department of Physiotherapy, Busia County Referral Hospital, Busia, Kenya; 2 Department of Physiotherapy, College of Health Sciences, Jomo Kenyatta University of Agriculture and Technology, Nairobi, Kenya; 3 Center for Research in Spinal Health and Rehabilitation Medicine, Department of Physiotherapy, College of Health Sciences, Jomo Kenyatta University of Agriculture and Technology, Nairobi, Kenya; 4 Department of Health and Rehabilitation Sciences, Stellenbosch University, Cape Town, South Africa

In the version of this article initially published, Mukoma, J.N., Matheri, J.M. & Tawa, N., 2020, ‘Prevalence and clinical characteristics associated with peripheral neuropathy amongst persons on HAART in Busia County, Kenya’, *South African Journal of Physiotherapy* 76(1), a1430. https://doi.org/10.4102/sajp.v76i1.1430, the affiliations for the third author, Nassib Tawa, was given incorrectly in the ‘Authors’ and ‘Affiliations’ sections. The author’s affiliations are now corrected as:

**Authors:**

John N. Mukoma^1^ https://orcid.org/0000-0003-1977-0366

Joseph M. Matheri^2^ https://orcid.org/0000-0001-9546-7006

Nassib Tawa^3,4^ https://orcid.org/0000-0003-2106-9384

**Affiliations:**

^1^Department of Physiotherapy, Busia County Referral Hospital, Busia, Kenya

^2^Department of Physiotherapy, College of Health Sciences, Jomo Kenyatta University of Agriculture and Technology, Nairobi, Kenya

^3^Center for Research in Spinal Health and Rehabilitation Medicine, Department of Physiotherapy, College of Health Sciences, Jomo Kenyatta University of Agriculture and Technology, Nairobi, Kenya

^4^Department of Health and Rehabilitation Sciences, Stellenbosch University, Cape Town, South Africa

This correction does not alter the study’s findings of significance or overall interpretation of the study’s results. The authors apologise for any inconvenience caused.

